# Signaling Proteins That Regulate Spermatogenesis Are the Emerging Target of Toxicant-Induced Male Reproductive Dysfunction

**DOI:** 10.3389/fendo.2021.800327

**Published:** 2021-12-24

**Authors:** Sheng Gao, Xiaolong Wu, Lingling Wang, Tiao Bu, Adolfo Perrotta, Giuseppe Guaglianone, Bruno Silvestrini, Fei Sun, C. Yan Cheng

**Affiliations:** ^1^ Department of Urology and Andrology, Sir Run Run Shaw Hospital, Zhejiang University School of Medicine, Hangzhou, China; ^2^ Institute of Reproductive Medicine, Nantong University School of Medicine, Nantong, China; ^3^ Department of Translational & Precision Medicine, Sapienza University of Rome, Rome, Italy; ^4^ Department of Hospital Pharmacy, “Azienda Sanitaria Locale (ASL) Roma 4”, Civitavecchia, Italy; ^5^ Institute of Pharmacology and Pharmacognosy, Sapienza University of Rome, Rome, Italy

**Keywords:** testis, spermatogenesis, endocrine disrupting chemicals, Sertoli cells, cytoskeletons, male reproduction

## Abstract

There is emerging evidence that environmental toxicants, in particular endocrine disrupting chemicals (EDCs) such as cadmium and perfluorooctanesulfonate (PFOS), induce Sertoli cell and testis injury, thereby perturbing spermatogenesis in humans, rodents and also widelife. Recent studies have shown that cadmium (e.g., cadmium chloride, CdCl_2_) and PFOS exert their disruptive effects through putative signaling proteins and signaling cascade similar to other pharmaceuticals, such as the non-hormonal male contraceptive drug adjudin. More important, these signaling proteins were also shown to be involved in modulating testis function based on studies in rodents. Collectively, these findings suggest that toxicants are using similar mechanisms that used to support spermatogenesis under physiological conditions to perturb Sertoli and testis function. These observations are physiologically significant, since a manipulation on the expression of these signaling proteins can possibly be used to manage the toxicant-induced male reproductive dysfunction. In this review, we highlight some of these findings and critically evaluate the possibility of using this approach to manage toxicant-induced defects in spermatrogenesis based on recent studies in animal models.

## 1 Introduction

An endocrine disrupting chemical (EDC) is an exogenous chemical, usually an environmental toxicant, capable of interfering with the function of endogenous hormones that are essential to maintain body function including reproduction, development, growth, metabolism, behavior and/or cell/tissue/organ homeostasis in humans and other mammals. In fact, the list of EDCs has been growing for the last decade due to industrial activities in which pharmaceutical industry synthesize new chemicals for their use in consumer products (1–3). These include plastic softeners (also known as plasticizers) for utensils and rubbers, stain- and stick-resistant chemicals to increase durability of clothing, fabrics, draperies, and vinyl flooring and wall covering products, as well as paints by modifying their components namely pigment, solvent and resin. These also include personal care products, such as nail polish, antiperspirants, deodorants, hair sprays, shampoos, soaps, and fragrance products. In fact, these chemicals are found in hundreds of products widely used by consumers across the globe. The EDCs include heavy metals (e.g., lead, mercury, cadmium), plasticizers (e.g., bisphenols, parabens, benzophenones, phthalates), and surfactants (e.g., PFOS, PFOA) (**Tables 1**, **2**) (3, 33–37). Studies have shown that many of these EDCs affects human reproductive function, pathogenesis of multiple diseases including carcinogenesis, obesity, diabetes, growth and development (in particular during fetal and child development) and others (37–39). For instance, recent studies have shown that exposure to EDCs is one of the major causes of idiopathic male infertility (40–43) and also reproductive dysfunction in females including primary ovarian insufficiency, endometriosis, preterm birth and earlier puberty (33, 43, 44).

**Table 1:** Effects of cadmium chloride (CdCl_2_) on male reproductive function*.

Species/cell line	Route of administration	Treatment dose	Observed effects	Refs
Human	*In vivo*	Natural environmental exposure	Sertoli cell injury; reduced inhibin B, AMH, and FSH in serum Reduced semen quality Reduced sperm concentration and motility Idiopathic oligoasthenozoospermic males had high levels of cadmium in serum, associated with impairment of sperm motility; higher sperm DNA fragmentation	(4–7)
Human sperm	*In vitro*	10 µM	Reduced sperm motility and DNA integrity; an increase in sperm DNA fragmentation and in intracellular oxidative stress in sperm	(8)
Human sperm	*In vitro*	20 μM	Reduced nucleus diameter; reduced mitochondrial membrane potential (MMP); an increase in DNA fragmentation.	(9)
Mouse	*In vivo*, i.p.	1 and 2.5 mg/kg b.w.	Reduced testis weight; reduced sperm survival and serum testosterone; an increase in sperm abnormality and ROS level.	(10)
Mouse	*In vivo*, i.p. *In vivo*, i.p. *In vivo*, oral	3 mg/kg b.w. 1.2 mg/kg b.w. 24 mg/kg b.w.	Germ cell exfoliation; Leydig cell degeneration; an increase in abnormal sperm morphology Seminiferous epithelial degeneration; Leydig cell injury; reduced serum testosterone; reduced Sertoli TJ function	(11, 12)
Mouse sperm	*In vitro*	14-55 µM	Reduced sperm motility	(13)
Rat	*In vivo*, oral *In vivo*, i.p. *In vivo*, i.p.	5 mg/kg b.w. 2 mg/kg b.w. 3 mg/kg b.w.	Reduced body weight, reduced sperm count, motility and viability; an increase in sperm defects; seminiferous epithelial degeneration Seminiferous epithelial injury; defects in spermatogenesis; intertubular haemorrahage; germ cell exfoliation Germ cell exfoliation; Sertoli cell injury with vacuoles found in cell cytosol; seminiferous epithelial disorganization	(14–16)
Rat Sertoli cell line	*In vitro*	1 µM	Sertoli cell injury; Sertoli cell tight junction disruption	(17)
Fish	*In vivo*, oral	5-40 µM	Germ cell exfoliation; germ cell injury with vacuoles in the cytosol of spermatogonia, spermatocytes, and spermatids; reduced semen quality	(18)
Chicken	*In vivo*, oral	140 mg/kg b.w.	Deformation of the seminiferous tubules; germ cell exfoliation	(19)

*This list is not intended to be exhaustive, it summarizes recent findings that illustrate effects of PFOS on male reproductive function based on studies in vivo and in vitro. AMH, anti-Mullerian hormone; FSH, follicle stimulating hormone; ROS, reactive oxygen species.

**Table 2:** Effects of perfluorooctane sulfonate (PFOS) on male reproductive function*.

Species/cells	Route of administration	Treatment dose(s)	Phenotypes	References
Human	*In vivo*	Natural environmental exposure	Subfertility; reduced sperm count; reduced sperm motility; an increase in serum levels of LH and FSH but reduced inhibin B Germ cell chromosomal aneuploidies and DNA fragmentation; reduced sperm quality and an increase in the population oc immature sperm	(20)(21, 22)
Human Sertoli cells	*In vitro*	20 and 40 μM	Sertoli cell injury manifested by truncated actin filaments and truncated microtubules across cell cytosol	(23)
Rat	*In vivo*, oral	0.5 - 6 mg/kg b.w.	Germ cell degeneration and testicle edema	(24)
Rat Sertoli cells	*In vitro*	20 μM	Sertoli cell injury manifested by truncation of actin filaments; TJ-barrier disruption; perturbed GJ communication	(25)
Mouse	*In vivo*, oral	0.5 or 10 mg/kg b.w.	An increase in germ cell apoptosis but reduced proliferation; reduced serum testosterone; reduced sperm count with vacuolations in spermatogonia, spermatocytes, and Leydig cells	(26)
Mouse	*In vivo*, oral	0.3 and 3 mg/kg b.w.	Low levels of adrenic acid and docosahexaenoic acid (DHA) in neonatal testes; reduced serum testosterone and reduced epididymal sperm count in postnatal mice	(27)
Mouse	*In vivo*, oral	0.5-10 mg/kg b.w.	Reduced sperm count in the epididymis and reduced serum testosterone; disrupted BTB and defects in spermatogenesis	(28)
Mouse Leydig cells	*In vitro*	15 and 30 mM	Reduced secretion of testosterone by Leydig cells due to defects in steroidogenesis	(29)
Mouse Sertoli cells	*In vitro*	5–60 μM	Disruption in Sertoli cell TJ-barrier function due to reduced expression of TJ and GJ proteins	(30)
Fish	*In vivo*	0.5 mM	Reduced sperm quality; structural defects in testis; an increase in serum E2; increased in estrogen receptor α1 levels	(31)
*C. elegans*	*In vivo*	0.375-10 mM	Reduced in germ cell population; reduced spermatid size and motility; an increase in spermatid defects	(32)

*This list is not intended to be exhaustive, it summarizes recent findings that illustrate effects of PFOS on male reproductive function based on studies in vivo and in vitro. BTB, blood-testis barrier, E2, estradiol-17ß; GJ, gap junction; TJ, tight junction.

More important, some EDCs have a very long half-life in humans. As such, high level of EDCs can be accumulated in the human body over an extended period of time, often years and also decades. For instance, cadmium has a half-life of >20 years (45, 46) *vs.* >5 years for PFOS in humans (47, 48). Furthermore, administration of a single EDC for a controlled study in rodents (or exposure of humans to a single EDC) may mask the physiological effects and its health risk since each animal (or person) is exposed to multiple EDCs simultaneously because of their widespread presence in our environment through foods, water, and air. Furthermore, selected life style, such as smoking, of the study subjects can also affect the outcome of a study. For instance, when laboratory animals were exposed to a mixture of phthalates in “dose addition model” and compared to results that obtained when toxicant was administered individually, the outcomes could be considerably different. Findings from the dose addition model studies have shown that a mixture of phthalates produce additive effects (49, 50), and the phenotypes are far worse than the sum of the combined individual effects. As such, changes that were found following low-dose exposure to a single EDC may not necessarily demonstrate the ‘real’ health risk. Thus, it is important to perform long-term studies using a combination of common EDCs to assess their health risks at doses that mimic the environmental (or industrial) exposure.

In this review, we focus our discussion based on recent reports on selected EDCs, namely cadmium and PFOS, that were found to perturb male reproductive function in particular spermatogenesis through putative signaling proteins and/or pathways. These findings are therapeutically important since the EDC-induced Sertoli cell or testis injury was shown to be blocked or rescued through an interference of the signaling proteins utilized by these EDCs in rodents (25, 51, 52), and in humans such as the use of primary human Sertoli cell cultures in studies (23, 53). As such, if these findings can be expanded in particular using other toxicants, some common signaling proteins and/or signaling cascades may be identified, in particular through the use of transcriptomics and pertinent omics including multiomics approaches. This information, in turn, can be helpful to alleviate toxicant-induced reproductive dysfunction. In brief, we narrowly focus on signaling proteins that are involved in EDC-induced Sertoli cell and/or testis injury based on studies in cadmium and PFOS. This approach is used since several eminent reviews on the larger topic of toxicant-induced reproductive dysfunction are found in the literature, many of which are cited here, to avoid redundancy.

## 2 Protein Kinases Capable of Alleviating EDC-Induced Sertoli Cell Injury in Rodents and/or Humans

### 2.1 Focal Adhesion Kinase (FAK)

#### 2.1.1 Background

**Figure 1:**
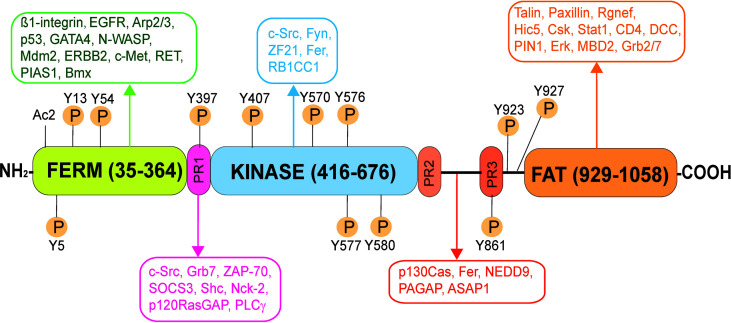
Schematic illustration of the human focal adhesion kinase (FAK). FAK in humans is a polypeptide composed of 1058 amino acid residues (NP_001186578.1), comprised of different functional domains. The different functional domains along the FAK polypeptide from its N-terminus are the FERM (band 4.1, ezrin, radixin, moesin homology) domain, followed by the catalytic kinase domain, and the FAT (focal adhesion targeting) domain near its C-terminus, and also three Pro-rich regions (PR1, PR2 and PR3). There are multiple putative phosphorylation sites including Tyr-397, -407, -576, -577, -861, and Y927, which are well conserved across species. Following its activation through phosphorylation, FAK serves as a signaling platform wherein different regulatory proteins (e.g., c-Src, c-Yes, Fyn, Fer, Erk, Csk) and adaptors (e.g., talin, paxillin) can bind to FAK. As such, FAK can recruit additional signaling and regulatory proteins to its different functional domains to modulate cellular functions, such spermatogenesis. EGFR, epidermal growth factor receptor; Arp2/3, actin-related protein 2/3 complex; p53, tumor protein p53; GATA4, GATA binding protein 4; N-WASP, neuronal Wiskott-Aldrich syndrome protein; Mdm2, mouse double minute 2 homology (also known as E3 ubiquitin-protein ligase, a regulator of the p53 tumor suppressor); ERBB2, Rrb-b2 receptor tyrosine kinase 2; c-Met, MET proto-oncogene, receptor tyrosine kinase; RET, rearranged during transfction, a proto-oncogene; PIAS1, protein inhibitor of activated STAT 1; Bmx, BMX non-receptor tyrosine kinase; Fyn, FYN proto-oncogene, c-Src, cellular Src transforming kinase; ZF21, zinc finger FYVE-type containing 21; RB1CC1, RB1 inducible coiled-coil 1; Grb7, growth factor receptor bound protein 7; Rgnef, Rho guanine nucleotide exchange factor 28; Hic5, transforming growth factor ß1 induced transcript 1; Csk, C-terminal Src kinase; Stat1, signal transducer and activator of transcription 1; DCC, DCC netrin 1 receptor; PIN1, peptidylprolyl cis/trans isomerase, NIMA-interacting 1; Erk, mitogen-activated protein kinase 1; MBD2, methyl-CpG binding domain protein 2; STAT1, signal transducer and activator of transcription 1; SRC, SRC proto-oncogene, non-receptor tyrosine kinase; ZAP-70, zeta chain of T cell receptor associated protein kinase 70; SOCS3, suppressor of cytokine signaling 3; Shc, SHC-adaptor protein; Nck-2, NCK adaptor protein 2; p120RasGAP, RAS p21 protein activator 1; PLCγ, phospholipase C γ1; p130Cas, BCAR1 scaffold protein, Cas family member; ASAP1, ArfGAP with SH3 domain, ankyrin repeat and PH domain 1; NEDD9, neural precursor cell expressed, developmentally down-regulated 9; Graf, GTPase regulator associated with FAK; Fer, FER tyrosine kinase.

**Figure 2:**
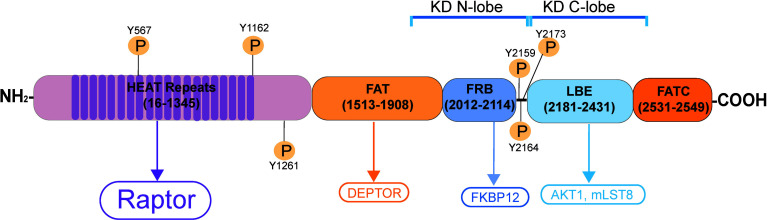
Schematic illustration of the human mTORC1 signaling complex. mTORC1 (mammalian target of rapamycin, NP_001373429.1) is a non-receptor Ser/Thr protein kinase, when it binds to its adaptor protein Raptor (regulatory-associated protein of mTOR), this creates the mTORC1 (mammalian target of rapamycin complex 1) signal complex. mTOR contains two distinctive catalytic domain (KD) containing intrinsic protein kinase activity called KD N-lobe (near the N-terminus) and the KD C-lobe (near the C-terminus). It has 20 tandem HEAT repeats near its N-terminus, to be followed by the FAT domain, FRB domain, LBE domain and the FATC domain near its C-terminus. Akt1; transforming retrovirus Akt1, an onocogene, also known as PKB (protein kinase B); C-lobe, C-terminal lobe; DEP, Dishevelled, Egl-10 and Pleckstrin; DEPTOR, DEP domain-containing mTOR interacting protein; EF3, elongating factor 3; FAT domain, FRAP, ATM, TRAP domain; FATC, FAT domain at the C-terminus; FKBP12, FK506/rapamycin-binding protein; HEAT, Huntington, EF3, PP2A, TOR1; FRAP, FKBP rapamycin associated protein; FRB, FKBP132 rapamycin binding; KD, kinase domain; LBE, binding site for mLST8; mLST8, mammalian lethal with SEC thirteen 8, also known as mTOR associated protein LST8 homolog; N-lobe, N-terminal lobe; PKB, protein kinase B; Raptor, regulatory-associated protein of mTOR, an adaptor protein.

**Figure 3:**
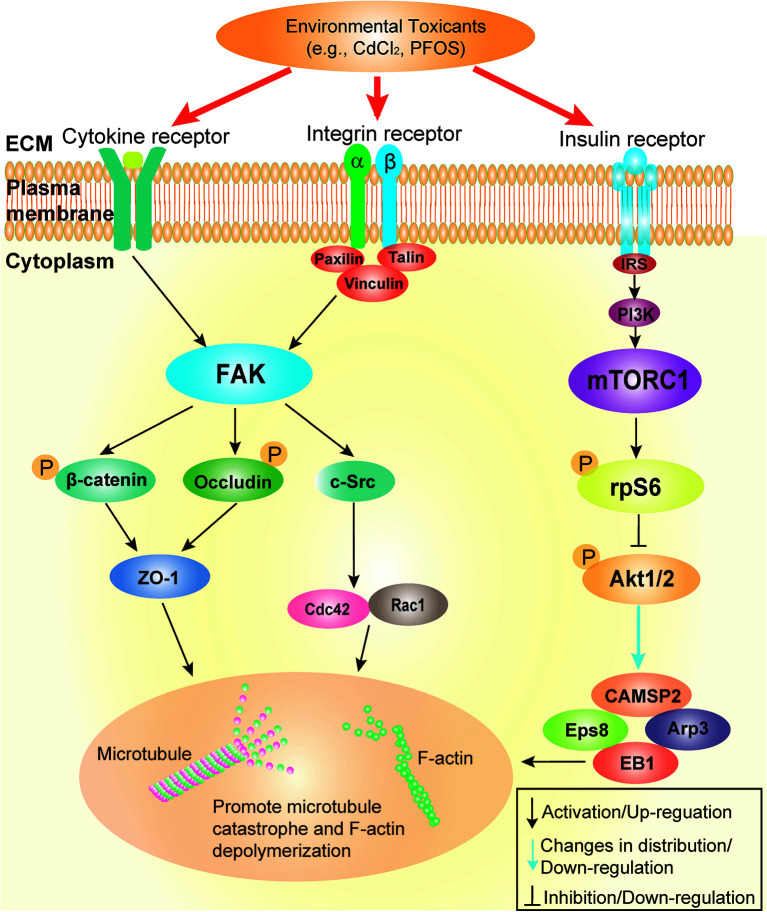
Schematic illustration by which cadmium and PFOS induce Sertoli cell and/or testis injury *via* FAK and mTORC1. This schematic illustration was prepared based on findings using environmental toxicant models of CdCl_2_ and PFOS. It is noted that the upstream ligand(s) utilized by cadmium and PFOS remain to be identified, but this likely involves either integrin-, cytokine- and/or insulin-based receptors. Both FAK and mTORC1 signaling protein/complex exert their corresponding effects downstream through different actin and microtubule (MT) regulatory proteins (*see text for details*). These changes, either through up- or down-regulation of the corresponding regulatory proteins, or distributions across the seminiferous epithelium, in turn, alter the structural organization of either actin- or MT-based cytoskeleton. The net result of these changes induce Sertoli cell or testis injury.

FAK is a known regulatory component of the focal adhesion complex (FAC, also called focal contact) at the cell-extracellular matrix (ECM or cell matrix) interface and an actin-based cell-matrix anchoring junction type (54, 55). FAK is also a crucial signaling protein that works in concert with integrins to relate integrin-based signaling cascade *via* its different interacting domains along its polypeptide sequence as shown in **Figure 1** (56). In fact, FAK is a prime target of anticancer therapy, being actively investigated by clinicians and scientists in recent years (57–59). FAK also involves in numerous cellular and physiological functions in cells and tissues, but also pathogenesis of diseases in particular carcinogenesis (57, 60). In the testis, FAK, however, is not found at the FAC since FAC is absent in the testis (61, 62). Instead, the only cell-matrix anchoring junction found in the testis is the intermediate filament-based hemidesmosome found at the base of the seminiferous epithelium, between basement membrane (a modified form of ECM in the testis) and the base of Sertoli cells (63, 64). Interestingly, FAK, and most notably its two activated/phosphorylated forms: p-FAK-Y397 (65, 66) and p-FAK-Y407 (67) (**Figure 1**) are constituent and regulatory component of the apical ES (apical ectoplasmic specialization) and basal ES (which together with tight junction (TJ) constitute the blood-testis barrier (BTB) in the testis), respectively. On the other hand, studies have shown that the robust expression of p-FAK-Y397 at the apical ES persists until late stage VIII tubules when the release of spermatozoa takes place near the edge of the tubule lumen (66, 68), suggesting it may be crucial to support spermatid adhesion at the apical ES. In fact, the α6ß1-integrin/p-FAK-Y397 complex is likely a crucial regulatory protein complex to modulate the release of sperm at spermiation (69–71). Interestingly, the use of a phosphomimetic mutant of p-FAK-Y397, namely p-FAK-Y397F, making it constitutively inactive was found to promote Sertoli cell TJ-barrier function making it tighter when overexpressed in the Sertoli cell epithelium with an established TJ-barrier (67). This is analogous to expressing p-FAK-Y407E (a phosphomimetic and constitutively active mutant of p-FAK-Y407) in Sertoli cells when its overexpression promoted the Sertoli TJ-barrier, making the barrier tighter (67). On the other hand, the use of a p-FAK-Y407F (a phosphomimetic and constitutively inactive mutant of p-FAK-Y407) was found to perturb the Sertoli cell TJ-barrier following its overexpression (67). Other studies have shown that FAK involves in maintaining the phosphorylation status of the cell adhesion proteins at the BTB/basal ES site, such as occludin (72). For instance, FAK determines whether these proteins (e.g., occludin) stay at the Sertoli cell-cell interface to support the TJ-permeability function of the BTB, or these proteins (e.g., occludin) at the BTB site should be internalized (55, 67, 72). This thus provides a novel mechanism to induce transient “opening” of the BTB to facilitate the transport of preleptotene spermatocytes across the barrier at stages VII-VIII of the epithelial cycle when p-FAK-Y407 robustly expresses at the BTB (67). Studies have shown that FAK, in particular p-FAK-Y397 and p-FAK-Y407 exert their regulatory effects at the corresponding apical ES and basal ES/BTB through changes at the F-actin and microtubule cytoskeletal organization (67, 73). Importantly, the use of a biochemical assay that monitors the ability of lysates of primary Sertoli cells cultured *in vitro*, overexpression of p-FAK-Y407E in cells transfected with pCI-neo/p-FAK-Y407E mutant vs. control cells transfected with pCI-neo, is able to enhance actin polymerization considerably (67). On the other hand, overexpression of a human p-FAK-Y407E phosphomimetic (i.e., constitutively active) mutant in human Sertoli cells is also capable of blocking the PFOS-induced MT defragmentation (23), such that MTs stretched across the entire human Sertoli cell cytosol, analogous to control human Sertoli cells (23). Collectively, these findings are consistent with earlier studies in fibroblasts, and epithelial and endothelial cells in which FAK is involved in actin and MT polymerization (74–78). In brief, these two activated/phosphorylated forms of FAK (and mTORC1, see **Figure 2**) appear to serve as molecular switch to turn the apical ES and BTB/basal ES “on” or “off”, depending on their expression status at the microdomain of these sites across the seminiferous epithelium (**Figures 2**, **3**).

#### 2.1.2 Studies of the PFOS and Cadmium Models, and the Adjudin Pharmaceutical Model

**Figure 4:**
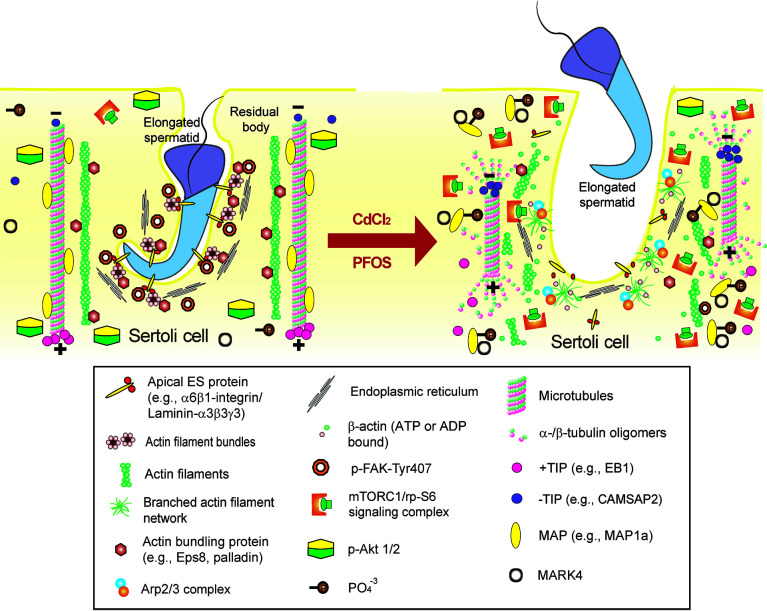
Environmental toxicant-induced testis injury through changes in germ cell adhesion, leading to germ cell exfoliation. *Left* panel depicts schematic illustration of intact seminiferous epithelium, such as in a stage VII-early VIII tubule, wherein intact apical ES supports spermatid adhesion to the Sertoli cell in the seminiferous epithelium during the final stage of spermiogenesis. The integrity of MT cytoskeleton is maintained by binding of MAPs (e.g., MAP1a) to the MT protofilaments and MT stability is also maintained by binding of the +TIP (e.g., EB1), and the proper level of –TIP (e.g., CAMSAP2) at its corresponding plus (+) and minus (-) ends. On the other hand, the actin cytoskeleton is maintained by binding of the actin bundling proteins (e.g., palladin and actin barbed end capping and bundling protein Eps8) to maintain actin filament bundles at the apical ES but also across the epithelium as aggregates of actin filaments to support Sertoli cell structural integrity. This microenvironment is also maintained by robustly expression of p-FAK-Y407 and likely p-Akt1/2. However, the unexpected presence of either toxicant (e.g., cadmium or PFOS) alter the microenvironment of the testis. For instance, a surge in MARK4 induces phosphorylation of MAP1a, causing their detachment from MTs, which is coupled with a down-regulation of +TIP (e.g., EB1) and more –TIP (e.g., CAMSAP2) binding to the corresponding ends of the MT. These changes thus destabilize MTs, leading to MT catastrophe. The expression and/or distribution of actin bundling proteins (e.g., palladin, Eps8) are also affected, these changes, coupled with a surge in Arp2/3 complex, causing “debundling” of actin filaments at the apical ES since actin filaments becoming a branched network due to an increase in actin branched nucleation induced by the Arp2/3 complex, thereby destabilizing F-actin network. The disruptive changes in the microenvironment include a considerable decline in p-FAK-Y407 and a surge in mTORC1 (with a concomitant down-regulation of p-Akt1/2) expression. The net result leads to premature germ cell exfoliation as noted in the *right* panel (*see text for details*), thereby perturbing male fertility due to defects in spermatogenesis.

**Figure 5:**
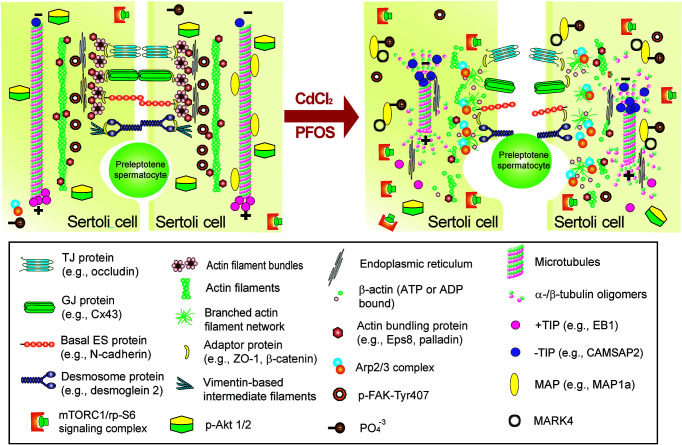
Environmental toxicant-induced testis injury through changes in Sertoli cell adhesion at the BTB, leading to defects in spermatogenesis. *Left* panel depicts schematic illustration of intact seminiferous epithelium near the basement membrane at the site of the BTB, such as in a stage VII tubule, wherein intact basal ES maintains BTB integrity to support spermatogenesis. The integrity of MT cytoskeleton at the BTB and across the seminiferous epithelium is maintained by binding of MAPs (e.g., MAP1a) to the MT protofilaments, and MT stability is also maintained by binding of the +TIP (e.g., EB1) and the proper level of –TIP (e.g., CAMSAP2) at its corresponding plus (+) and minus (-) ends. On the other hand, the actin cytoskeleton is maintained by binding of the actin bundling proteins (e.g., palladin and Eps8) to maintain actin filament bundles at the basal ES/BTB but also across the epithelium as aggregates of actin filaments to support Sertoli cell structural integrity. This microenvironment is also maintained by robust expression of p-FAK-Y407 and likely p-Akt1/2. However, the unexpected presence of cadmium or PFOS alters the microenvironment of the testis at the BTB but also across the seminiferous epithelium. For instance, a surge in MARK4 induces phosphorylation of MAP1a, causing MAP detachment from MTs, which is coupled with a down-regulation of +TIP (e.g., EB1) and more –TIP (e.g., CAMSAP2) binding to the corresponding ends of the MT. These changes thus destabilize MTs, leading to MT catastrophe. The expression and/or distribution of actin bundling proteins (e.g., palladin, Eps8) are also affected, these changes, coupled with a surge in Arp2/3 complex, causing “debundling” of actin filaments at the basal ES/BTB since actin filaments becoming a branched network due to an increase in actin branched nucleation induced by the Arp2/3 complex, thereby destabilizing F-actin network. This disruptive change in the microenvironment is maintained by a considerable decline in p-FAK-Y407 and a surge in mTORC1 (with a concomitant down-regulation of p-Akt1/2). The net result leads to a loss of BTB integrity, making the barrier more permeable to toxicants/biomolecules as noted in the *right* panel (*see text for details*), thereby perturbing male fertility due to defects in spermatogenesis.

The initial report that illustrates p-FAK-Y407 is involved in PFOS-induced Sertoli cell injury was first published in 2014 (52). This study showed that PFOS-mediated disruption of the Sertoli TJ-barrier function associated with a considerable down-regulation of p-FAK-Y407, but not p-FAK-Y397. Furthermore, PFOS also considerably perturbed the cytoskeletal organization of F-actin across the Sertoli cell epithelium with an established functional TJ-barrier, in which actin filaments were considerably truncated and disorganized, analogous to the phenotypes when Sertoli cells were transfected with a specific FAK miRNA (called miR-135b, which is known to knockdown FAK (52, 79, 80). This report thus establishes the likely mechanism by which PFOS induces Sertoli cell injury, likely through a down-regulation of p-FAK-Y407, which in turn, perturbs the Sertoli cell F-actin organization (52) (**Figures 4**, **5**). This notion is also supported by an earlier report which has shown that p-FAK-Y407 indeed promotes Sertoli cell BTB function by stabilizing actin cytoskeleton at the Sertoli cell epithelium with a functional BTB *in vitro* (67). Interestingly, it was shown that overexpression of a phosphomimetic (and constitutively active) mutant of p-FAK-Y407, namely p-FAK-Y407E, in the Sertoli cell epithelium *in vitro*, was capable of blocking the PFOS-induced Sertoli cell injury by re-establishing the PFOS-mediated Sertoli cell TJ-barrier disruption (52). Overexpression of p-FAK-Y407E was also capable of inducing proper re-organization of the actin cytoskeleton which was perturbed by PFOS (52), likely through an increase in actin polymerization kinetics (67). An earlier report has also shown that FAK is capable of recruiting occludin to the Sertoli cell-cell interface, possibility through changes in the phosphorylation status of occludin (72). These changes, in turn, promoted proper distribution of the occludin-ZO-1 complex at the Sertoli cell-cell interface, thereby re-establishing the PFOS-induced disrupted TJ-barrier (52). More important, these findings were recently confirmed in primary Sertoli cell cultures *in vitro* in which PFOS not only perturbed the organization of actin- but also microtubule (MT)-based cytoskeletons (23). For instance, overexpression of a human p-FAK-Y407E phosphomimetic (and constitutively active) mutant was shown to re-establish the PFOS-induced human Sertoli cell TJ-barrier disruption, which is the result of a proper re-organization of actin and MT cytoskeletons across the human Sertoli cell epithelium (23). Collectively, these findings are important because they have unequivocally demonstrated for the first time that by manipulating a putative signaling protein, namely p-FAK-Y407 whose expression was down-regulated by PFOS, such as through overexpression of a phosphomimetic and constitutively active mutant p-FAK-Y407E, both in rodents and humans, the PFOS-induced Sertoli cell injury can be alleviated.

A recent report based on studies *in vivo* has also shown that cadmium chloride (CdCl_2_)-induced testis injury (3 mg/kg b.w., *via* i.p.) was remarkably notable by as early as 6 hr by assessing changes in the organization of microtubule cytoskeleton across the seminiferous epithelium (81), consistent with more recent studies (82). This is also the time when the phenotypes across the seminiferous epithelium were not distinctively noted by histological analysis (81). Besides extensive truncation and disorganization of the MT protofilaments across the seminiferous epithelium, considerable disruption regarding distribution of the MT regulatory proteins MAP1a and CAMSAP2 were also notably detected (81). These findings are physiologically significant since MAP1a (microtubule associated proteins 1a, a MAP) is known to bind onto the microtubules to confer MT stability (83). On the other hand, CAMSAP2 (calmodulin-regulated spectrin-associated protein 2) is a microtubule minus (-) end targeting protein (-TIP) known to confer MT dynamics, determining the length of the MT protofilaments in response to changes in cellular environment (84), such as at different stages of the epithelial cycle in the testis. These findings suggest that toxicant-induced testis or Sertoli cell injury is more than a simple general cell toxicity cellular event, but involving a well defined toxicant-induced cascade of events including signaling and regulatory proteins. More important, these are the same signaling proteins that are being used by the testis to support testis function and spermatogenesis, illustrating that toxicants exert their toxic effects through defined signaling pathways. In this context, it is of interest to note that toxicants, including cadmium, also exert their disruptive effects in the testis through an increased oxidative damage in the seminiferous epithelium involving FAK (82, 85). It is obvious that much work is needed to relate these findings to p-FAK-Y407 in the cadmium model and to delineate the precise underlying molecular mechanism(s). The current concept by which PFOS and cadmium induce Sertoli cell and testis injury is summarized in **Figures 4**, **5**. Additionally, in parallel experiments that investigated changes in cytoskeletal organization and the downstream signaling proteins using the pharmaceutical model adjudin, we have discovered a remarkable negative correlation between p-FAK-Y407 expression and the adjudin-mediated defects in spermatogenesis in the testis *in vivo* (81). In this context, it is of interest to note that adjudin is a non-hormonal male contraceptive known to induce extensive germ cell exfoliation (86, 87) by primarily targeting the actin and microtubule cytoskeletons in the Sertoli cells across the seminiferous epithelium (81, 87–89). It has also been used to serve as a model to study Sertoli cell-cell and Sertoli-germ cell adhesion and BTB dynamics in the testis, making this a novel model to decipher the underlying mechanism(s) that regulate spermatogenesis (89–92). Using this pharmaceutical adjudin model, we have detected extensive disruption of the MT cytoskeletal organization across the seminiferous epithelium, which tightly associated with a considerable down-regulation of p-FAK-Y407 in testes (81). This finding is also consistent with earlier findings in which the spatiotemporal expression of p-FAK-Y407 in normal testes is tightly associated with the BTB in the testis, and overexpression of p-FAK-Y407E phosphomimetic (and constitutively active) mutant in Sertoli cell epithelium promotes BTB function making it “tighter” (67). On the other hand, p-FAK-Y407 is robustly expressed at the apical ES to confer spermatid adhesion onto Sertoli cells in the seminiferous epithelium, but its expression is remarkably reduced at the Sertoli cell-spermatid interface (i.e., apical ES) in late stage VIII when the release of sperm occurs (67). Collectively, these data thus indicate that findings obtained from the use of toxicant and/or pharmaceutical models are consistent with physiological data using normal testes *in vivo*. These changes also highlight the importance of delineating the downstream signaling proteins and the involving signaling cascade utilized by toxicants to induce male reproductive dysfunction. Since this information will be crucial to provide new approaches to manage toxicant-induced reproductive dysfunction and idiopathic infertility, and other pathological conditions, such as carcinogenesis.

### 2.2 mTORC1/rpS6/Akt1/2 Signaling Complex

#### 2.2.1 Background

Studies have shown that the mTORC1 (mammalian target of rapamycin complex 1)/rpS6 (ribosomal protein S6)/Akt1/2 (transforming thymic lymphomas Akt1/2 kinase, also known as PKB, protein kinase B, a non-receptor Ser/Thr protein kinase) complex is one of the most prevalent signaling protein complexes that regulates cellular metabolism found in virtually all mammalian cells (93–95). mTORC1 is created by binding of the mTOR (mammalian target of rapamycin, a Ser/Thr non-receptor protein kinase) and its adaptor protein Raptor (regulatory-associated protein of mTOR) (**Figure 2**) (94). Besides energy metabolism in mammalian cells, mTORC1/p-rpS6/p-Akt1/2 is an emerging regulatory signaling complex crucial to support spermatogenesis through its effects on Sertoli cell function (95, 96) (**Figure 3**). The role of mTORC1 (and mTORC2) in regulating mammalian spermatogenesis in studies of the rat and mouse has been recently reviewed (95, 96), so additional discussion is not included here to avoid redundancy. Nonetheless, studies using genetic models have also confirmed our earlier findings indicating the significance of both mTORC1 and mTORC2 in testis functions (97). For instance, Sertoli cell-specific deletion of mTOR led to infertility in mice, which associated with a surge in p-rpS6 expression, loss of Sertoli cell polarity, increased in germ cell apoptosis and extensive germ cell exfoliation, but also gap junction impairment due to mislocalization of connexin 43 (Cx43) (98). On the other hand, germ cell-specific deletion of mTOR also led to infertility in mice even though these mice were viable and apparently healthy (99). In adulthood, spermatogonial stem cells in these mice failed to proliferate and differentiate into spermatocytes to undergo meiosis, and seminiferous tubules were devoid of virtually all germ cells, leading to infertility (99). These findings also illustrate that these mTOR deletion mediated infertility is more than just defects in energy metabolism since developing germ cells rely on Sertoli cells for their energy needs. As such, specific deletion of mTOR in germ cells should not interfere with their energy metabolism since Sertoli cells remain in the testis to support their metabolic and nutritional needs. Studies have shown that this mTORC1-based signaling complex exerts its effects by modulating cytoskeletal organization of actin and MT in Sertoli cells across the seminiferous epithelium, involving changes in energy metabolism but also intrinsic activities and/or distribution of regulatory proteins of both cytoskeletons (95, 96). In brief, it is increasingly clear that the mTORC2 complex modulates cytoskeletal function by activating mTORC1 initially, to be followed by a surge in the expression of p-rpS6-S235/S236 and p-rpS-S240/S244, and then a down-regulation of the p-Akt1-S473 and p-Akt2-S474 expression (**Figure 1**) as noted in two reports (100, 101). Interestingly, this signaling complex has been shown to be involved in PFOS- and adjudin-mediated Sertoli cell or testis injury.

#### 2.2.2 Studies of the PFOS and the Pharmaceutical Adjudin Models

Using the PFOS model in Sertoli cells cultured *in vitro* with an established TJ-permeability barrier, this environmental toxicant was found to perturb both actin and MT cytoskeletons through a down-regulation of the p-Akt1-S473 and p-Akt2-S474 (51). For instance, actin filaments that stretched across the entire Sertoli cell cytosol noted in control cells were considerably truncated and mis-aligned, no longer stretching properly across the Sertoli cell to support cell function, which in turn perturbed the TJ-barrier function (51). On the other hand, MT protofilaments that stretched across the entire Sertoli cell cytosol in control cells to support cell function were also considerably altered. For instance, MTs were considerably shortened and wrapped around the Sertoli cell nuclei instead (51). In order to confirm that PFOS exerts its disruptive effects to induce Sertoli cell injury is indeed involving p-Akt1/2, we used a specific Akt1/2 activator, namely SC79 (2-amino-6-chloro-α-cyano-3-(-ethoxycarbonyl)-4H-1-benzopyran-4-acetic acid ethyl ester, Mr 364.78) for the study. It is known that SC79 binds to the plecktrin homology (PH) domain of the Akt polypeptide, mimicking the binding of PtdIns(3,4,5)P3 to Alt to induce conformational change by enhancing its phosphorylation and activation at p-Akt1-T308 and p-Akt1-S473 sites (102). As anticipated, SC79 indeed was capable of blocking PFOS-mediated Sertoli cell injury by alleviating the PFOS-mediated Sertoli TJ-barrier disruption through corrective changes in the proper organization of actin- and MT-based cytoskeletons across the cell cytosol (51). In a recent *in vivo* study using the pharmaceutical adjudin model, we also reported a remarkable surge in the expression of p-rpS6-S235/S236 and p-rpS6-S240/S244 following treatment of adult rats with a single dose of adjudin at 50 mg/kg b.w. (oral gavage) (81). This observation is also consistent with an earlier study, reporting a surge in expression of mTOR and p-rpS6 (but not total rpS6) following treatment of adult rats with adjudin (single dose, 250 mg/kg b.w., oral gavage) (103). Collectively, these findings together with the data summarized and discussed in *Section 2.1* have provided compelling evidence that environmental toxicants exert their effects by disrupting key signaling proteins, most notably their phosphorylation status, which are necessary and essential to support spermatogenesis and testis function under physiological conditions (**Figures 3**–**5**). Furthermore, new activator(s) of p-Akt1/2, instead of SC79, with reduced cytotoxicity should be carefully evaluated in future studies.

## 3 Concluding Remarks and Future Perspectives

As discussed above, we have provided a critical summary on the two signaling proteins that are being used by the toxicants cadmium and POFS to induce male reproductive dysfunction, through changes in their phosphorylation status (**Figures 3**–**5**). In order to expand this work further prior to studies *in vivo*, it may be advantageous to develop an *in vitro* human testis model that mimics the testes *in vivo* for toxicity studies. This *in vitro* human testis model should have the capability of developing functional haploid spermatids from spermatogonical stem cells (SSCs), mimicking the testis *in vivo*. The use of such a model thus reduces the time it takes to translate findings from studies in rodents to humans. There are much interest in the field to develop polymer- or hydrogel-based microfluidic devices for tissue engineering that mimic ion channels in human cells/tissues, bone tissue regeneration for transplantation, and perhaps tubules found in kidneys, prostate and others (104–108). A major obstacle of developing an *in vitro* human testis model is that the polymer- or hydrogel-based microfluidic device, unlike the seminiferous tubules in the testis, is not a dynamic structure. Since these hydrogel- or biopolymer-based devices are not capable of undergoing disassembly, reassembly, and dynamic maintenance, similar to the actin- and MT-based cytoskeletons in the seminiferous tubules, in response to changes of the epithelial cycle during spermatogenesis in the testis. Nonetheless, it is our beliefs that such an *in vitro* human testis can be established in the near future due to advances in cell/tissue cultures and engineering technology. On the other hand, it remains to be established if these EDCs, such as cadmium and PFOS, exert their disruptive effects directly, such as through direct changes on the phosphorylation status and/or distribution of the involving signaling proteins, or indirectly, such as through a down-regulation of the enzymes that change the testosterone availability through steroidogenesis. These possibilities should also be carefully evaluated in future studies.

## Author Contributions

CYC conceived the project and wrote the paper. SG, XW, LW, TB, and CYC researched on the topics and searched for relevant information in the literature at www.PubMed.com and relevant journal sites, which were discussed and cited in this review. SG and CYC prepared the Tables. SG and CC prepared the figures. SG, XW, LW, TB, AP, GG, BS, and FS discussed the concepts evaluated in this review. All authors contributed to the article and approved the submitted version.

## Funding

This work was supported in part by grants from the National Key Research and Development Program of China (2018YFC1003500 to FS), and Fellowships from the Noopolis Foundation (Rome, Italy, to XW, LW, TB).

## Conflict of Interest

The authors declare that the research was conducted in the absence of any commercial or financial relationships that could be construed as a potential conflict of interest.

## Publisher’s Note

All claims expressed in this article are solely those of the authors and do not necessarily represent those of their affiliated organizations, or those of the publisher, the editors and the reviewers. Any product that may be evaluated in this article, or claim that may be made by its manufacturer, is not guaranteed or endorsed by the publisher.
